# A Hidden Crisis in Nursing Homes: Examining Dementia Confidence and Negative Job Experience

**DOI:** 10.1093/geroni/igaf122.3689

**Published:** 2025-12-31

**Authors:** Joonyoung Cho, Angela Perone

**Affiliations:** University of Hawaiʻi at Mānoa, Honolulu, Hawaii, United States; University of California, Berkeley, Berkeley, California, United States

## Abstract

While dementia care needs in nursing homes are increasing, staff positions are often unfilled. Research suggests that some workers report challenging experiences by residents (e.g., communication problems, disrespectful behaviors). Confidence in providing care might shape their experiences, yet we know little about whether confidence in providing dementia care is associated with negative experiences. To address this gap, we used data from the National Dementia Workforce Study Nursing Home Survey Wave 1, an annual survey of dementia workers in the US. Our final sample size was restricted to respondents who provided information on confidence and negative experiences with residents (N = 374). Confidence in providing dementia care is coded as a continuous variable by summing the following 4 dimensions: (1) use past information to improve communication, (2) adapting working style for tailored care (3) keep motivating, and (4) play an active role in the team. Two separate multivariate logistic regressions revealed that confidence in providing dementia care is associated with lower odds of experiencing communication difficulties with residents (OR = 0.70, p ≤ 0.001). In contrast, confidence in providing dementia care is not associated with experiencing disrespectful behaviors from residents (OR = 0.85, p = 0.176). This study suggests that interventions that build confidence in dementia care (e.g., targeted training), including strategies for navigating communication and behavior challenges, could improve dementia care worker experiences and strengthen this workforce overall.

